# Calcined Clays as Concrete Additive in Structural Concrete: Workability, Mechanical Properties, Durability, and Sustainability Performance

**DOI:** 10.3390/ma17184517

**Published:** 2024-09-14

**Authors:** Bastian Strybny, Tobias Schack, Julian Link, Michael Haist

**Affiliations:** Institute of Building Materials Science, Leibniz Unviversity Hannover, 30167 Hannover, Germany; t.schack@baustoff.uni-hannover.de (T.S.); j.link@baustoff.uni-hannover.de (J.L.); haist@baustoff.uni-hannover.de (M.H.)

**Keywords:** calcined clays, structural concrete, workability, mechanical, durability, sustainability

## Abstract

Calcined clays (CCs) as supplementary cementitious materials (SCMs) can be a promising option to reduce clinker content and CO_2_ emissions in eco-friendly concretes. Although CCs as components of composite cements in combination with Ordinary Portland Cement (OPC) and limestone powder (LSP) have attracted industry interest, their use as concrete additives is limited. This study investigates the effects of the addition of CCs on the fresh and hardened properties of industry-standard ready-mixed concretes. Four concrete mix designs, each with three superplasticizer dosages, were tested, resulting in 12 variations. The CCs used, which are typical of 2:1 bentonite clays with low metakaolin content, reflect the clays available in Germany. The results showed that CCs significantly influenced the workability, which could be controlled with a high superplasticizer dosage. Increased CC contents reduced bleeding tendencies, which was beneficial for certain structural applications. Early age strength decreased with CCs, but the 28-day strength exceeded that of pure OPC concretes up to 30 wt% CCs. Resistance to CO_2_-induced carbonation decreased with higher levels of CCs but was comparable up to 15 wt%. Freeze–thaw damage decreased, and chloride migration resistance improved due to a denser microstructure. The global warming potential (GWP) of the concretes tested is in line with that reported in the literature for concretes made from highly blended cements, suggesting that CCs can improve the sustainability of concrete production.

## 1. Introduction

In order to realize the CO_2_-reduction targets in the building materials and construction sector, various approaches have been proposed and are currently being implemented. The majority of these approaches focus on the substitution of Ordinary Portland Cement (OPC) clinker for supplementary cementitious materials (SCMs) [1]. This can be done either on the binder level by replacing OPC with binary or ternary composite cements [2,3,4] or on the concrete level by reducing the (composite) cement content and adding, e.g., fly ash or limestone powder (LSP) to the mixture [5]. Both approaches, however, are compromised by the fact that the availability of chemically reactive SCMs, such as fly ash or blast furnace slag, is significantly declining [6]. The calcined clays (CCs) discussed here are proposed as a viable alternative, as they contribute to strength formation in the concrete by their pozzolanic reactivity [6,7,8,9].

The physical properties of CCs, as well as their chemical reactivity, are significantly influenced by the calcination and milling process. Compared to standard OPCs, it can be generally stated that CCs possess an increased specific surface and reduced particle size [10]. As a consequence, the water demand to ensure proper workability of concretes with CCs increases [8,9,10,11,12]. CCs with a high kaolinite content led to a disproportionate increase in water demand, which is reflected in a significant reduction in workability. Without adjusting the water content, a substitution of 15 wt% of OPC by metakaolin, e.g., has been shown to reduce the spread flow of concrete by up to 90% [13]. In contrast, the high content of smectite clays has a negligible influence on workability [14]. Generally, 1:1 clays have shown a higher water demand than 2:1 clays due to the different layer structure [15,16].

If the water addition or the dosage of admixtures is not adjusted, the increased water demand results in reduced workability of the fresh concrete and increased Bingham yield stress and plastic viscosity, resulting in reduced pumpability, air void formation due to insufficient filling of the formwork and increased compaction effort [17]. Similar to OPC mixes, an increase in the water content to compensate for the poor workability may lead to increasing porosity in the hardened concrete, thus requiring suitable admixtures to ensure workability [18,19,20].

The addition of polycarboxylate ether (PCE)-based superplasticizer generally shows a liquefying effect on CCs; however, a pronounced thixotropy—i.e., shear history-dependent changes in the dynamic viscosity of such concretes—are dominant [21]. The interaction of superplasticizers with CCs is highly influenced by the mineralogical composition of the CCs [22]. Investigations show a higher demand for PCE dosage for calcined kaolinite-rich clays than for calcined phyllosilicates-rich clays [23]. The availability of montmorillonites can cause chemical sorption of the PCE and result in a loss of the dispersing effect of the superplasticizer [24]. To reach proper workability with CCs, the dosage of the PCE must be increased by a factor of 4 to 6 [12]. Generally, it is essential to adjust the dosage of the superplasticizer to the overall system and especially to the composition of the CC. 

Despite the undesirable effects on concrete workability, the decreasing flowability with the addition of CCs has been shown to reduce the risk of bleeding and sedimentation in fresh concrete. Significant reductions in bleeding and sedimentation processes were achieved with a cement substitution rate of 20 wt% of CCs by weight of the binder [25]. 

The pozzolanic reaction also reduces the porosities due to the consumption of Ca(OH)_2_ and the precipitation of C-(A)-S-H-phases, which result in the formation of a denser microstructure and thus the development of greater resistance to chemical attacks [8,26]. The compressive strength of concrete increases by up to 140% due to lower porosities, depending on the substitution rate and the composition of metakaolin [27]. Commonly, concretes with CCs show higher compressive strengths with the onset of the pozzolanic reaction [8,9].

With regard to durability, the reduced porosity causes an increased resistance against chloride migration. With the substitution of OPC by CCs, lower chloride migration coefficients were determined for low-grade kaolinitic clay [28,29]. However, the consumption of Ca(OH)_2_ results in a decrease in the pH value, which reflects in a more rapid carbonation. Investigations with a low-grade CC show a constantly increasing carbonation front penetration with an increasing cement substation rate [30].

With regard to the freeze–thaw resistance of concrete, the composition of CCs has shown to be decisive, as concretes with metakaolin show a high resistance and concretes with calcined illitic or montmorillonitic clays show a lower resistance to freeze attacks than concretes with a binder consisting of OPC [29]. Generally, it can be said that the resistance to freeze–thaw attacks decrease with CC content. 

The described influences on the fresh and hardened state concrete properties due to the addition of CCs require close attention to ensuring concrete workability. In order to counteract the increased water demand, the dosage of admixtures should be carefully adjusted to maintain the application-related flowability without promoting sedimentation processes. The increase in viscosity due to higher cohesion of the CCs can be counteracted by reducing the fine aggregate [31]. However, this step must be regarded as very delicate, as a change in the grading curve influences the entire rheology and the packing density in the hardened concrete. In individual international research projects, substitution rates of 20 wt% for metakaolin and 50 wt% for CCs in combination with limestone powder were achieved for selected clays [31]. A comprehensive investigation of possible substitution rates for concretes with clays available in Europe or in Germany has not yet been carried out and represents a missing step towards implementing the use of CCs in practical, sustainable concretes. 

Research activity on the usage of CCs in concrete primarily focussed either on so-called LC3 systems, a closely defined mixture of high metakaolin CCs, LSP and OPC [6,32,33,34]. Increasing research activity was also presented on 2:1 clays [16,35]. However, these approaches all have a primary focus on the development of new composite cements in common. However, studies on the use of CC as an additive in concrete are also available in the literature [8,9,10,11,12]. In the opinion of the authors, this approach also harbors great potential for the use of CC as an additive in concrete production, e.g., replacing fly ash with diminishing availability. The main challenge here is that in contrast, e.g., to the LC3 system where the individual binder components were carefully adjusted to each other, when used as an additive, e.g., in the ready-mixed plant, CCs face a much broader bandwidth of interactions. The paper at hand explicitly focuses on such an application in structural concrete. It investigates the influence of different substation rates of OPC by a low-grade CC—typical for central Europe—on the fresh and hardened state concrete properties. The focus of this study is not only on the mechanical properties but also on the durability-relevant properties of structural concrete in central Europe, e.g., carbonation and chloride migration resistance. In addition, the sustainability performance of the concretes with CC as an additive is assessed. Contrary to the general trend of using clinker-reduced cements, an OPC was used as a reference binder to avoid the influence of other additives in composite cements.

## 2. Materials and Methods

### 2.1. Raw Materials

In the presented investigations, an Ordinary Portland Cement CEM I 42.5 R (HeidelbergCement AG, Plant Ennigerloh, Heidelberg, Germany) and a 2:1 CC from a German manufacturer (Liapor GmbH & Co. KG, Plant Pautzfeld, Hallerndorf, Germany) were used as binders. The CC has a low metakaolin content of approximately 32% and, therefore, low reactivity [18,36]. As for the aggregates, river sand and gravel (Mineral Baustoff GmbH, Plant Tündern, Bad Hersfeld, Germany) with a maximum grain size of 16 mm were used. 

The physical properties of the binders, as well as the oxide compositions, are detailed in Table 1 and Table 2. Both the OPC and the CC were provided by the Priority Program DFG SPP 2005 project program, “Opus Fluidum Futurum—Rheology of reactive, multiscale, multiphase construction materials”. Detailed information on the binders can be found in [37]. 

### 2.2. Concrete Mix Design

A series of 12 different concrete compositions were tested in this study, varying the ratio of CC content to the total binder content (mass CC + mass OPC) and the dosage of superplasticizer. Instead of adding the CC as part of the cement, it was added as an additive in the mixer. The binder compositions were prepared by gradually reducing the amount of OPC by weight in steps of 100%, 85%, 70%, and 50% and adding CC accordingly. The resulting mixes are designated accordingly as C100-CC0, C85-CC15, C70-CC30, and C50-CC50. Although replacing 50 wt% of cement with calcined clay is not practical for concrete applications, this study is intended to also show the limits. However, 15 wt% and 30 wt% CC are also used, for example, for other additives such as fly ash. The total binder content in the concrete was set to 360 kg/m^3^, corresponding to strength class C30/37 according to DIN EN 1992-1-1 [38], which is typical for the German ready-mixed market. The water/cement w/c or water/binder w/b ratio was chosen to be 0.5 for all mixes. The aggregate size distribution corresponded to a grading curve A/B according to DIN 1045-2 [39], with a maximum grain size of 16 mm. All of the aggregates were used in dry conditions. 

To adjust the flow properties of the fresh concrete, three different dosages of a polycarboxylate-based superplasticizer (Master Builders Solution, MasterGlenium SKY 594) were examined for each mix. The dosages were selected to achieve three different consistency classes according to DIN 1045-2 [39] for the OPC concrete (C100-CC0). The defined amounts of superplasticizer were 1.0, 1.35, and 1.80 wt% of binder and kept constant for each mix, resulting in a total number of 12 concrete compositions. Table 3 shows the detailed concrete compositions.

In addition to the main investigations, 12 concretes with constant superplasticzer contents, and only varying w/b values were produced to show the pure effect of the high water demand from the calcined clay.

A pan mixer (Zyklos, Pemat Mischtechnik GmbH, Freisbach, Germany) was used for mixing. First, the dry materials were mixed for 30 s. Then, water and superplasticizer were added within 30 s and mixed for an additional 180 s until a homogeneous fresh concrete was achieved. The mixing procedure is detailed in Table 4.

### 2.3. Test Methods

The flowability of the fresh concrete was evaluated using the flow table test following DIN EN 12350-5 [40], 10 min after the first water–binder contact. The air void content of the fresh concrete was determined using the pressure gauge method by DIN EN 12350-7 [41], and the density was measured in the same test vessel (5 dm^3^) according to DIN EN 12350-6 [42].

The bleeding test, known as the “bucket” test, was conducted in accordance with the methodology described in [43]. However, in contrast to the procedure described in [43], the bucket was filled in two distinct layers and subsequently compacted on a vibrating table with a constant compaction energy of 4500 rpm for 15 s for each layer. At 30 min intervals (starting 30 min after the mixing process), the surface water of the freshly poured concrete was collected using a syringe, weighed, and then returned to the concrete. The measurement was considered complete when there were no changes in the concentration of water on the surface. The quantity of water separated per m^3^ of concrete can be calculated based on the sample mass and the measurement of the density.

The compressive strength of the samples was quantified following the specifications of DIN EN 12390-3 [44] at 1, 7 and 28 days of age. The samples were maintained within their molds for a period of 24 h, after which they were demolded, submerged under water and kept at a stable ambient temperature of 20 ± 2 °C prior to testing.

To examine the resilience of the material to carbonation induced by carbon dioxide, an accelerated carbonation test in accordance with the standards set out in DIN EN 12390-12 [45] was performed. The samples for testing were removed from the mold after 24 h and were then kept in the water reservoir at 20 ± 2 °C for a period of 27 days. Subsequently, the samples were maintained in a climate chamber at a temperature of 20 ± 2 °C and a relative humidity of 65 ± 3% for a period of 42 days. Thereafter, they were subjected to an elevated CO_2_ concentration of 3.0 ± 0.5 vol% for a further 28 days. In order to investigate the carbonation depth of the concrete, the specimens were split along the length axis, and the visible cross-section was treated with phenolphthalein. The depth of carbonation was measured at three points from each of the four sides of each slice, commencing with the outermost point. For each sample, the mean carbonation depth was calculated from the 12 resulting measuring points.

The rapid chloride migration (RCM) test was performed to determine the chloride migration resilience, following the procedure outlined in [46]. Cylindrical samples with a diameter of 100 ± 1 mm and a height of 50 ± 5 mm were prepared and placed in the water reservoir at 20 ± 2 °C after 24 h until the time of testing at 35 days. The RCM tests were performed using a 0.2 N potassium hydroxide solution (KOH) as the anode and a 0.2 KOH + 10% NaCl solution as the cathode. To assess the chloride migration, the cylindrical samples were divided into two parts along the vertical axis. Initially, a fluorescein solution was applied to the surface of the visible cross-section, followed by a silver nitrate solution. The depth of chloride penetration was in situ measured at 11 points from the bottom. However, the average value for each sample was calculated from nine measurements, with the two outermost points excluded. The chloride migration coefficient D_CI_ was computed using the methodology outlined in [46], utilizing the average values and the experimental boundaries criteria.

The CIF test, as described in [47], was carried out to assess the freeze–thaw resistance without the use of de-icing salt. Cube-shaped samples of 150 × 150 × 150 mm^3^ were used to measure capillary suction and internal and outer damage over a period of freeze–thaw cycles. Following a 24 h period in the mold, the samples were demolded and stored in a water reservoir at a temperature of 20 ± 2 °C for 6 days. After that, new samples measuring 150 × 100 × 70 mm^3^ were extracted from each cube using a saw. Subsequently, the newly prepared samples were subjected to a period of dry conditions, maintained at a temperature of 20 ± 2 °C and a relative humidity of 65 ± 3%, within the climate chamber up to the age of 28 days. Following this, the samples were exposed to a 7-day single-sided water storing period, during which their capillary suction behavior was quantified. The study involved determining the relative dynamic modulus of elasticity as a sign of internal damage as well as the concrete spalling as a sign of outer damage over 28 freeze–thaw cycles (12 h per cycle).

## 3. Experimental Results

### 3.1. Fresh Concrete Properties

**Figure 1:**
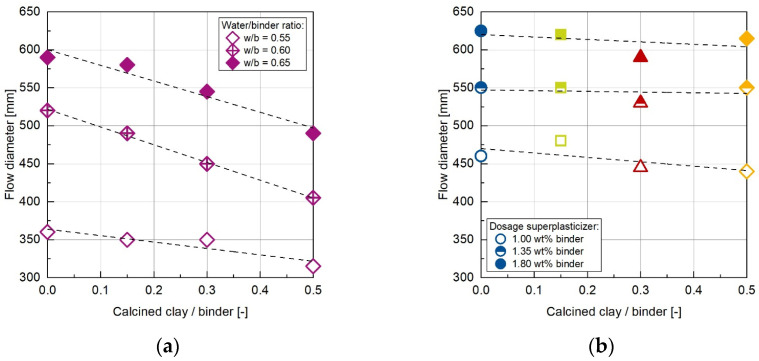
(**a**) Changes of the concrete spread flow diameter in dependence of the CC content for concretes without superplasticizer but with different water–binder ratios; (**b**) for concretes with various superplasticizer dosages and constant water–binder ratios w/b = 0.5.

The substitution of cement by CC changes the physical properties of the binder, which has a major influence on the flow properties of the concrete. The consistency of the concrete decreases with increasing CC content, as shown in Figure 1a. This tendency is valid for all investigated water/binder ratios and is caused by the higher specific surface of the CC compared to the OPC. Maintaining workability is an essential challenge when using CCs in concrete. The addition of superplasticizers changed the consistency from the former consistency class F2 without superplasticizer to F3, F4, and F5 (DIN 1045-2 [39]) with the corresponding superplasticizer. As can be seen from Figure 1b, the detrital effect of CC onto the concrete spread flow could be completely compensated for by the addition of a superplasticizer.

**Figure 2:**
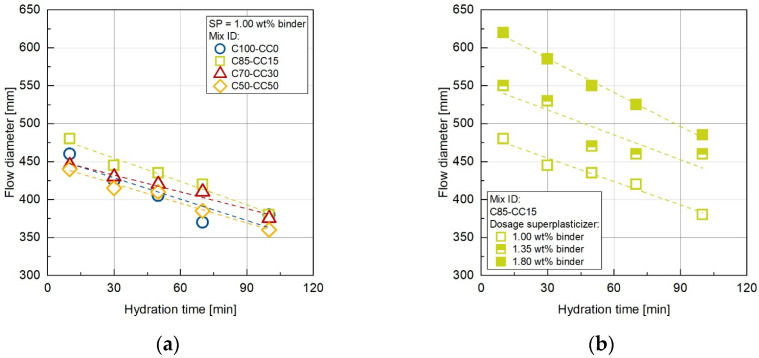
(**a**) Changes of the concrete spread flow diameter in dependence of the hydration time for different CC contents with superplasticizer dosages of 1.00 wt% per binder; (**b**) for a selected mix with a CC content of 15 wt% with various superplasticizer dosages.

Figure 2a shows the changes in concrete spread flow diameter for different concrete ages (i.e., durations after water addition) for all the mixes for a selected superplasticizer dosages of 1.00 wt% per binder. As expected, a reduction in the flow diameter occurs with increasing age for all binder combinations. Figure 2b gives the influence of different superplasticizer dosages for the selected mix with a CC content of 15 wt%, clearly showing that by adjusting the dosage superplasticizer, the flow diameter can be closely set. The loss of consistency over time is only slightly influenced by the superplasticizer dosage, as can be seen from the almost parallel regressions. However, the superplasticizer dosage of 1.00 wt% resulted in a consistency decrease with flow diameter reductions of around 100 mm within the investigated time steps. The highest superplasticizer dosage of 1.80 wt% caused the maximum decrease in flowability due to hydration, with a reduction of flow diameter values by 135 mm. With regard to the effect of changing binder compositions by adding CC, it can be stated that the impact of CC on the concrete workability becomes less significant when superplasticizers are used, as already shown in Figure 1. One possible reason for this could be the adjustment of the superplasticizer dosage to the binder content. Assuming that the superplasticizer polymers have a preference for interacting with cement particles instead of CC particles due to the higher chemical affinity of the cement, reducing the cement content then replacing it with CC would result in an increase in the proportion of superplasticizer per cement content [16,48]. For the pure OPC concrete (C100-CC0), a superplasticizer dosage of 1.00 wt% per binder content is equivalent to a dosage of 1.00 wt% per cement content. In comparison, for the C50-CC50 concrete, the same dosage of 1.00 wt% per binder is available, but twice the amount per cement, meaning 2.00 wt% per cement content. The reduced workability caused by the addition of CCs to the concrete composition can be mitigated by adjusting the superplasticizer dosage to the effective cement content [12]. 

**Figure 3:**
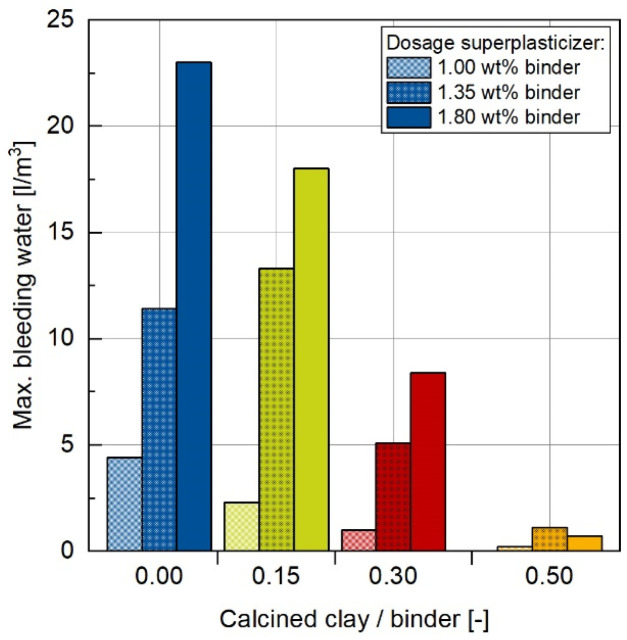
Changes in the maximum bleeding water are dependent on the CC content for concretes with different superplasticizer dosages.

The bleeding tendency of all concrete mixes is shown in Figure 3. It is evident that both the proportion of CC in the concrete composition and the dosage of superplasticizer have an impact on the amount of bleeding water. The smallest changes in bleeding tendency due to the addition of CCs to the system were detected for the lowest superplasticizer dosage of 1.00 wt%. Therefore, the maximum bleeding water content in the OPC C100-CC0 concrete only reached 4.4 L/m^3^ and can be reduced by CC addition to almost 0 L/m^3^ for the C50-CC50. However, significant differences in bleeding tendency due to CC substitution are observed for the highest superplasticizer dosage of 1.80 wt%. In that case, the maximum bleeding water could be reduced from values of 23 L/m^3^ in the C100-CC0 mixture to 0.7 L/m^3^ for the C50-CC50. The values of the 1.35 wt% superplasticizer dosage are in the middle range and confirm the described trend. Similar study results on the influence of CC on bleeding behavior were described in [23].

In addition to consistency and bleeding water tests, the air void content and fresh concrete density were also measured. The air void content decreases with increasing workability and ranges from 0.5 vol.-% for C100-CC0 to 1.1 vol.-% for C50-CC50. The fresh concrete density decreases with an increase in CC content. This can be explained by the lower powder density of CCs compared to the OPC (cf. Table 1).

### 3.2. Compressive Strength

**Figure 4:**
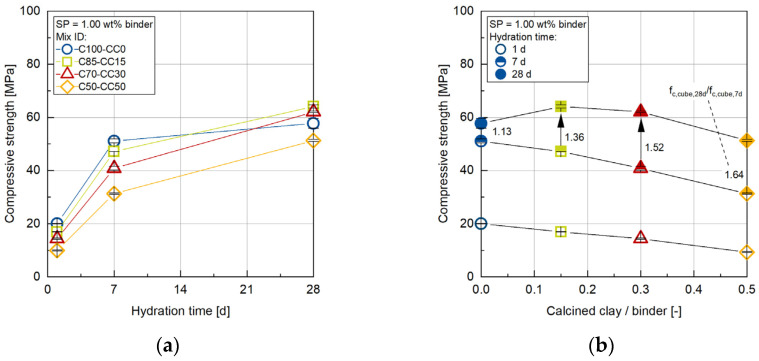
(**a**) Compressive strength of all concrete compositions investigated as a function of age; (**b**) compressive strength as a function of CC content (numbers and arrows indicate the factor by which the strength increased between 7 d and 28 d).

The compressive strength evolution of all concrete compositions is presented in Figure 4. In the early stage of hydration after 1 day, the higher the proportion of CC, the lower the compressive strength due to the lower reactivity of the CC [1]. The compressive strength of the C50-CC50 and C70-CC30 concretes is reduced by 28% and 53%, respectively. With a substitution rate of only 15 wt% CC, this results in a comparable value of 16.9 MPa compared to the OPC system with 20.0 MPa. After 7 days, the compressive strengths show comparable values to the OPC system, having the highest compressive strength of 51.1 MPa (C100-CC0) and the system with the highest substitution rate of 50 wt% CC having the lowest value of 31.3 MPa (C50-CC50).

At later ages of 7 days and 28 days, a greater increase can be found in the compressive strength in the mixes with CC as opposed to the OPC system (C100-CC0). With a substitution rate of 15 wt% (C85-CC15) and 30 wt% (C70-CC30) CC, the compressive strength after 28 days is higher than with the OPC system (C100-CC0). The prolonged pozzolanic reaction of CC contributes to this development [6,7,8,9]. The calculated ratio between the compressive strength after 28 days and 7 days (*f*_c,cube,28_/*f*_c,cube,7d_) can be used to estimate the proportion of pozzolanic reaction during this period (cf. Figure 4b). The OPC system (C100-CC0) shows a value of *f*_c,cube,28_/*f*_c,cube,7d_ = 1.13. In contrast, the concretes with CC have significantly higher values of *f*_c,cube,28_/*f*_c,cube,7d_, which increase with increasing CC content from 1.36 (C85-CC15) to 1.64 (C50-CC50). Looking at this high value of *f*_c,cube,28_/*f*_c,cube,7d_ or the gradient of compressive strength evolution in the period from 7 days to 28 days, it can be considered that the compressive strength of the concrete with 50 wt.% CC (C50-CC50) has a higher compressive strength than the OPC system (C100-CC0), at a later age. After 28 days, this value of 51.3 MPa is still less than that of the OPC system with 57.1 MPa.

### 3.3. Carbonation Resistance

**Figure 5:**
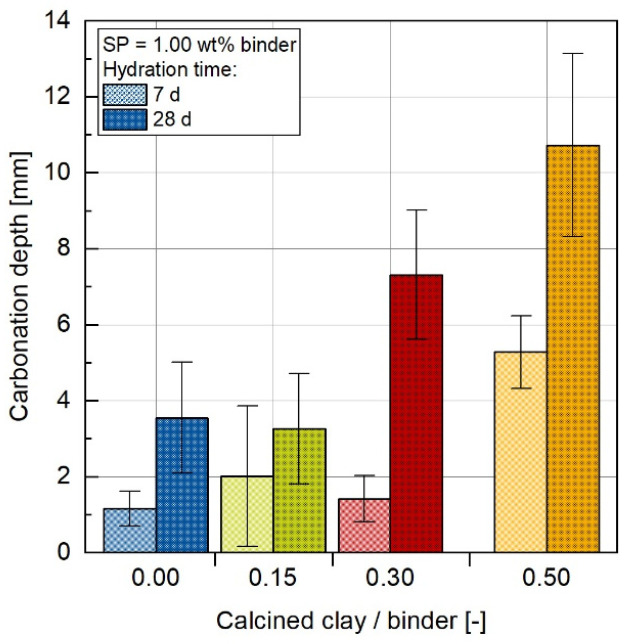
Changes of the carbonation depth in dependence on the CC content after 7 d and 28 d concrete age according to [45].

In order to determine the resistance to carbonation, the accelerated carbonation test was performed [45]. The carbonation depth changes as a function of the CC content to binder content ratio for samples aged 7 and 28 days, as shown in Figure 5. It is evident that an increase in CC content leads to greater carbonation depths. The C50-CC50 concrete is the most critical, with an average carbonation depth of 10.73 mm after 28 days. The carbonation intensity in concrete mixtures generally increases as the proportion of OPC clinker decreases. It is remarkable that the concrete mixture containing 15 wt% CC (C75-15CC) exhibits a slightly lower carbonation depth of 3.26 mm compared to the pure OPC concrete (C100-CC0), which has a carbonation depth of 3.55 mm. At this point, however, the influence of scattering must also be taken into account, as the measured average values lie within the range of the standard deviation according to 7 d, so that the two results do not differ statistically. The concrete mixture with 30 wt% CC (C70-CC30) with a depth of 7.31 mm confirms that trend.

### 3.4. Chloride Migration Resistance

**Figure 6:**
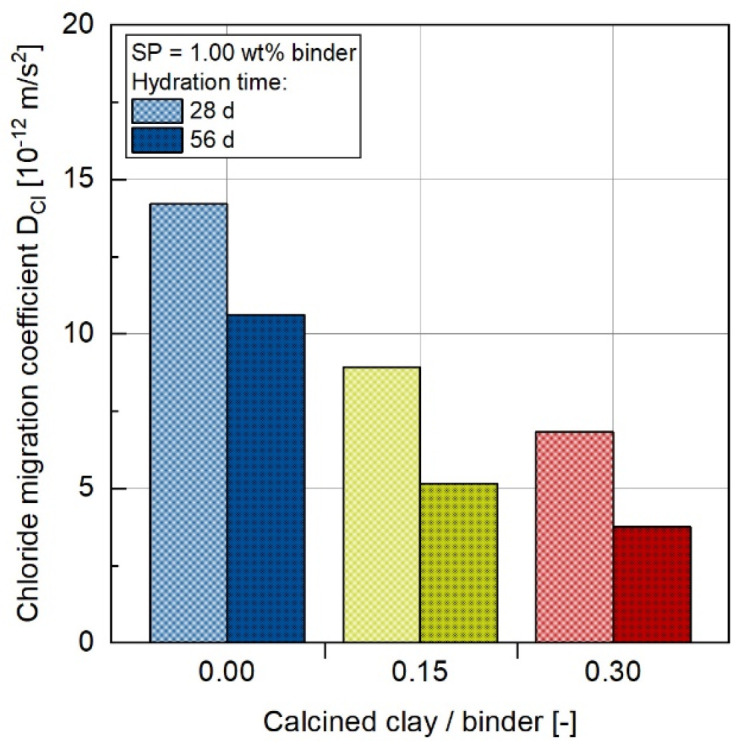
Chloride migration coefficient D_Cl_ determined with the RCM test [46].

Figure 6 presents the values of the chloride migration coefficient (D_Cl_) for concrete compositions with a CC content of up to 30 wt.%. The measurements of the rapid chloride migration test were carried out at 28 and 56 days of age. It can be clearly stated that the penetration of chloride ions into these concretes depends on the presence of CC. Concrete made only with OPC (C100-CC0) shows the highest chloride migration coefficient of 14.2·10^−12^ m^2^/s and thus the lowest resistance to chloride penetration. The coefficient decreases almost linearly with increasing CC content to 8.9·10^−12^ m^2^/s (C85-CC15) and 6.8·10^−12^ m/s^2^ (C70-CC30) at an age of 28 days. The pozzolanic reaction reduces the porosity due to the consumption of Ca(OH)_2_ and the precipitation of C-(A)-S-H-phases, which cause the formation of a more dense microstructure and thus the development of a greater resistance to ion transport [4,21]. The values of the concrete with CC are below the acceptance criterion of 10.0·10^−12^ m^2^/s according to [46] for exposure classes XS2 and XD2.

Regardless of the binder composition, the chloride penetration resistance increases with increasing hydration time. Accordingly, all concretes show low coefficients after 56 days compared to the measurements after 28 days. The decrease is at a comparable level for all concretes. The value of the concrete with a CC content of 30 wt% is 3.7·10^−12^ m/s^2^ (C70-CC30) below the acceptance criterion of 5.0·10^−12^ m^2^/s according to [46] for exposure classes XS3 and XD3.

### 3.5. Freeze–Thaw Resistance

**Figure 7:**
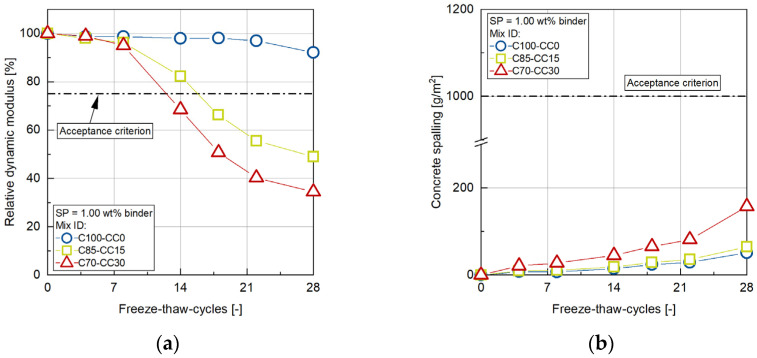
(**a**) Internal damage—relative dynamic modulus of elasticity; (**b**) and outer damage—concrete spalling—of all concretes investigated with the CIF-test according to [47].

To estimate the freeze–thaw resilience, the capillary suction behavior, internal damage via the dynamic modulus of elasticity, and outer damage via concrete spalling were measured as a function of a number of freeze–thaw cycles (FTC). The test was conducted at 28 days of age, starting with a 7-day capillary suction period. Figure 7 shows the change of the relative dynamic modulus of elasticity (a) and the concrete spalling (b) as a function of FTCs for the concretes C100-CC0, C85-CC15 and C70-CC30. It can be seen that the OPC system has a very high freeze–thaw resistance—after 28 freeze–thaw cycles, only a slight reduction in the relative modulus of elasticity to 92.14% can be observed. In contrast, the concretes with CC exhibit a significant decrease in the relative modulus of elasticity after 28 freeze–thaw cycles. The sharpest decline is found for the concrete with 30 wt% CC (C70-CC30) to a value of 34.40% after 28 freeze–thaw cycles. Even the concrete with a low substitution rate of 15 wt% CC (C85-CC15) has a low value of 48.90% for the relative modulus of elasticity after 28 freeze–thaw cycles. The values of the concrete with CC do not fulfill the acceptance criterion of 75% after 28 freeze–thaw cycles according to [47] for sufficient freeze–thaw resistance. In terms of concrete spalling, the OPC and the C85-CC15 are very close to each other. Only the C70-CC30 shows increased spalling. Nevertheless, all the concretes investigated here are well below the acceptance criterion of 1000 g/m^2^ according to [47], although the spalling test is only decisive for the CDF test with de-icing salt and not the here performed CIF test without de-icing salt.

### 3.6. Sustainability Assessment

Global Warming Potential (GWP) is the most important environmental impact of cement-based materials and is primarily used for sustainability assessment of concrete. The environmental impact resulting from the production of 1 m^3^ of concrete can be calculated by multiplying the impact resulting from the production of each raw material by the amount of raw materials used in the concrete and adding the individual impacts. The GWP can be computed using Equation (1), where n_i_ is the mass of material required to produce 1 m^3^ of concrete and p_i,CO2,e_ is the CO_2_ generated in the production of each raw material (Table 5).

GWP_CO2,e_ = ∑n_i·_p_i,CO2,e_ (1)

The GWP calculations in this study did not include the environmental impact of transporting raw materials to the concrete plant.

**Figure 8:**
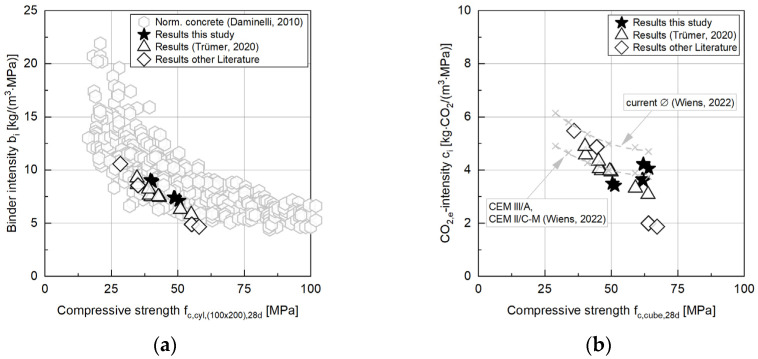
(**a**) Binder intensity b_i_; (**b**) CO_2,e_ intensity for the results from this study compared to the literature data [8,50,51].

In order to assess the ecological performance of the concrete, a combined analysis of the GWP data and the mechanical performance of the concretes was carried out. Figure 8a shows the binder intensity *b_i_* of the concrete compositions with CC in comparison to normal concretes (data according to [50]). The binder intensity *b_i_* of normal concretes—i.e., the amount of reactive binder that must be used to achieve 1 MPa in 1 m^3^ of concrete—increases disproportionately with decreasing compressive strength [50]. Comparing the results of the investigated concretes (i.e., containing CCs) with the data of ordinary concretes according to [50], it can be clearly seen that the binder intensity is at the lower limit of the ordinary concretes due to the use of CC as an additive in the binder. This is in line with comparable concretes with CC in the literature (cf. Figure 8). However, the overall trend is that the binder intensity increases by decreasing the compressive strength of the concrete, which remains the same for the concretes with CC.

Referring the calculated GWP values of the concretes to the compressive strength, it was found that the normalized GWP values of the concretes investigated with CC are at a comparably low level with concretes with highly blended cements (CEM III/A or CEM II/C-M—data according to [51]). The concretes examined in this study are well classified among comparable concretes with CC from the literature. All concretes with CC are below the current average of *c_i_* in Germany (data according to [51]).

## 4. Conclusions

The investigations carried out in this study support the potential for the use of CC as an additive for the production of structural concrete. The results indicate that CC contents of up to 30 wt% yield similar mechanical properties as pure OPC mixes. In addition, CC as an additive is particularly suitable for concreting applications in chloride-laden environments. However, the resistance to CO_2_ penetration and the freeze–thaw resistance decrease when CC is used as an additive, which makes the concretes analyzed in this study very suitable for indoor concrete elements with low durability requirements. A major focus of the investigations in this study was the evaluation of the influence of CC on workability.

Based on the results presented in this study, the following conclusions can be summarized:

- The decrease in workability resulting from the CC addition to the concrete composition can be compensated by adjusting the superplasticizer dosage.
- The bleeding tendency of the concretes is reduced with increasing CC dosage, even at high superplasticizer dosages.
- The air void content and bulk density of fresh concrete are not significantly affected.
- The early age strength is decreased for CC concretes, but the final strength after 28 days is improved for CC dosages up to 30 wt% (C70-CC30) compared to the pure OPC system (C100-CC0).
- The resistance against carbonation is generally reduced with increasing CC content, even though a carbonation resistance equivalent to that of OPC was found for a mix with 15 wt% CC.
- Internal damage due to freeze–thaw cycles is increased, so the exposure of the structural concrete element must be taken into account.
- The chloride migration resistance is strongly improved with increasing CC dosage due to the reduced porosity and denser microstructure.
- The GWP values of the concrete investigated are comparable to those of concretes with high blended cements.

Regarding other potential applications, in addition to those mentioned above, the inclusion of CCs as SCMs can also have a favorable effect on the aesthetic properties of concrete surfaces. This is due to the significantly reduced tendency of bleeding water, as shown in Figure 3. However, it is important to note that a higher proportion of CCs in the mixture can also change the color of the hardened concrete to a browner hue.

## Figures and Tables

**Table 1:** Physical characteristics of the binder components.

Component	Density	Specific Surface Area (Blaine Method)	Specific Surface Area (BET Method)	Particle Size		
				d_10_	d_50_	d_90_
	[g/cm^3^]	[cm^2^/g]	[m^2^/g]	[μm]		
CEM I 42.5 R	3.14	3499	1.04	1.92	14.10	41.49
CC	2.62	12,929	6.26	1.00	5.32	26.71

**Table 2:** Oxide composition of the binder components measured by the XRF method.

Component	CaO	SiO_2_	Al_2_O_3_	Fe_2_O_3_	MgO	K_2_O	Na_2_O	TiO_2_	P_2_O	Mn_2_O_3_	SO_3_	LOI	Cl^−^	Sum
	[wt%]													
CEM I 42.5 R	61.79	21.14	5.53	2.27	1.39	0.77	0.77	0.21	0.14	0.03	2.84	2.48	0.03	98.94
CC	5.25	52.57	21.69	8.12	2.35	3.02	0.33	1.04	0.27	0.09	0.78	3.84	-	99.35

**Table 3:** Concrete compositions with three dosages of superplasticizer per mix (w/b = 0.50).

Mix ID	Cement	CC	Water	Aggregates			w/b	w/c	CC_mass_	Fresh Concrete Density
				0/2	2/8	8/16			CC_mass_ + OPC_mass_	
	[kg/m^3^]	[kg/m^3^]	[kg/m^3^]	[kg/m^3^]			[-]	[-]	[-]	[kg/m^3^]
C100-CC0	360	0	180	621.3	550.3	650.4	0.50	0.50	0	2.40
C85-CC15	306	54	180	619.6	548.7	648.6	0.50	0.59	0.15	2.40
C70-CC30	252	108	180	617.8	547.2	646.7	0.50	0.71	0.30	2.37
C50-CC50	180	180	180	615.4	545.1	644.2	0.50	1.0	0.50	2.36

**Table 4:** Mixing procedure.

Time	Step Description	Duration
[s]		[s]
0–30	Mixing all dry components—aggregates, OPC and CC	30
31–45	Addition of water	15
46–60	Addition of superplasticizer	15
61–240	Mixing	180

**Table 5:** Global warming potential data of raw materials.

Raw Material	GWP	Source ^(1)^
	[kg·CO_2equ_./kg]	
CEM I 42.5 R	0.798	[49]
CC	0.150	[49]
Aggregate (0–16 mm)	0.00296	[49]
Water	0.000256	[49]
Superplasticizer	0.944	[49]

^(1)^ A broad literature review was carried out in [49], including publications on raw material information and common databanks (e.g., ÖKOBAUDAT).

## Data Availability

The original contributions presented in the study are included in the article, further inquiries can be directed to the corresponding author.
